# Associations of genetic variants at TAP1 and TAP2 with pulmonary tuberculosis risk among the Chinese population

**DOI:** 10.1017/S0950268821000613

**Published:** 2021-03-19

**Authors:** Mingwu Zhang, Xiaomeng Wang, Yelei Zhu, Songhua Chen, Bin Chen, Zhengwei Liu

**Affiliations:** Zhejiang Provincial Center for Disease Control and Prevention, Binsheng Road, Binjiang District, Hangzhou City, Zhejiang Province 310051, China

**Keywords:** Haplotype, molecular microbiology, pulmonary tuberculosis, single nucleotide polymorphism, transporter-associated antigen processing

## Abstract

Tuberculosis (TB) is a common infectious disease, and the present study aims to explore the associations of single nucleotide polymorphisms (SNPs) at rs1135216 and rs1057141 of transporter-associated antigen processing (TAP1) and rs2228396 of TAP2 with pulmonary tuberculosis (PTB) risk. A case–control study including 168 smear-positive PTB cases and 251 controls was conducted. Genotyping of the SNPs at rs1135216, rs1057141 and rs2228396 was performed, and their associations with PTB risk were analysed with SPSS software version 19.0. After conducting stratification for age, a significant association was detected for rs1057141 with increased PTB risk (OR = 0.17, 95% CI 0.04–0.79) among those aged ≥60 years. For those aged <60 years, a marginally significant association was detected between rs1135216 TC/CC and PTB risk (OR = 1.97, 95% CI 0.93–4.19). Haplotype analysis revealed that the haplotype AT at rs1135216 and rs2228396, as well as AAT at rs1057141, rs1135216 and rs2228396, was associated with increased PTB risk, and the ORs were 2.83 (95% CI 1.30–6.14) and 2.89 (95% CI 1.34–6.27), respectively. Rs1057141 is a genetic predictor of reduced PTB risk for those aged ≥60 years, while rs1135216 might be a potential genetic predictor for those aged <60 years. Haplotype AT at rs1135216 and rs2228396, as well as AAT at rs1057141, rs1135216 and rs2228396, is a genetic marker that may predict PTB risk.

## Introduction

Tuberculosis (TB) is a chronic infectious disease, and it was reported that 10.0 million new cases were detected and 1.2 million deaths from TB occurred among HIV-negative people in 2018 [1]. It has been estimated that one-third of the whole population is infected with *Mycobacterium tuberculosis* (MTB); 10% of these will develop TB while the other 90% may never develop symptomatic disease. Previous studies revealed that TB is a complex disease that is affected by many factors, including genetic variants, nutrition status and environmental factors, as well as interactions between the host and pathogen.

As a disease that is caused by a pathogen, pulmonary tuberculosis (PTB) is partly regarded as an immune-related disease. The incidence and development of PTB are associated with host immune status, which is mostly decided by host immune regulation-related genes [2], especially those responsible for cellular immunity, such as Toll-like receptor (TLR) family genes (which play roles in the recognition of exogenous material), as well as immune regulation-related cytokine family genes [3], inflammation response mediators, etc. Thus, host susceptibility studies mainly focused on the related genes and their interactions with the pathogen in recent years. As vital regulators for MTB recognition and immune activation, TLRs are regarded as critical elements for TB infection. Previous studies revealed that single nucleotide polymorphisms (SNPs) in TLR family genes were associated with TB risk [4–6], and downstream genes were implicated too, such as tumour necrosis factor [7], interleukins [8], etc.

Transporter-associated antigen processing (TAP) is responsible for dealing with exogenous pathogenic microorganisms and then handling and presenting them to immune cells [9]. Consequently, some mutations located within key areas of TAP genes can lead to MHC class I deficiency and the reduction of CD8^+^ T cells. Some genetic variants within TAP genes were found to be associated with cervical intraepithelial neoplasia [10] as well as haematological malignancies [11]. In recent years, Sunder *et al*. found that the SNPs at position 637 of TAP1 were associated with HIV-TB co-infection [12]. Other studies were performed on TAP polymorphisms and TB in various populations, including Koreans, Chinese, etc. [4, 13, 14]; however, the study conclusions were mostly inconsistent. Notably, the associations among the Chinese population needed to be clarified, and the present study aims to elucidate and verify the potential correlations.

Previous studies revealed that age is a vital factor of TB incidence, and ageing may lead to a continuous increase in the risk of TB [15]. Gender is considered to be another factor implicated in TB incidence, and TB is more common in males than females [16]. In order to clarify the impact of genetic factors on TB risk in different age and gender groups, the present study performed an analysis of the stratification to provide more clues for further study.

## Methods

### Ethics statement

The study was approved by the ethics committee of Zhejiang Provincial Center for Disease Control and Prevention, and written informed consents were obtained.

### Study population

A population-based case–control study in Changshan and Jiangshan counties in Zhejiang province was conducted. In the present study, 168 newly diagnosed smear-positive PTB cases were recruited from 1 January 2016 to 30 June 2017. Meanwhile, 251 clinically diagnosed non-TB patients or healthy controls were enrolled in the study from the same hospital, and all the controls were verified to never previously have TB.

All recruited subjects were instructed to complete a structured questionnaire with demographic information as well as life style-related questions including smoking, drinking, dietary habits, etc. Health-related information of the recruited subjects was also collected.

### Genotyping

Genotyping of SNPs at rs1057141and rs1135216 was analysed by polymerase chain reaction-restriction fragment length polymorphism. The primer sequences and the restriction endonuclease utilised, as well as their product sizes, are shown in Table 1.

**Table 1.** Primers adopted for genotyping of the three SNPs

SNPs	Primers	Sequences (5′–3′)	Restriction endonuclease	Products
rs1135216	F0	GAGTGCGTGAATGCATGAGT	AccI	97 + 249
	R0	GGGAACTGAGAACTGCAAGG		
rs1057141	F1	ACGTCCACCCTGAGTGATTC	Fba I	123 + 242
	R1	ATGTTGAGCAACCTGGGAAC		
rs2228396	P1	ACGTTGGATGCCTTATCATCTTCGCAGCTC		
	P2	ACGTTGGATGTGCACAGGTGGTTTCAGTTG		
	P3	AGCTCTGCAGCCCATAAG		

Each 10 μl PCR sample contained 30 ng of the DNA template, 5 μl of 2X Prime Taq Premix (Genet Bio, Korea), 2 mM of each primer and the appropriate amount of ddH_2_O. The PCR cycling parameters were set as follows: DNA denaturation at 95 °C for 5 min followed by 35 cycles of annealing at 95 °C for 30 s and extension at 72 °C for 30 s, with a final extension of 72 °C for 10 min. Three μl of PCR product was incubated at 37 °C with 3 U of each endonuclease, 4 μl of 2X buffer and the appropriate amount of ddH_2_O, and was then separated onto 2.5% agarose gels. The SNP of rs2228396 was detected by the MassARRAY® MALDI-TOF System. The primer sequences employed are shown in Table 1. Five per cent of the DNA samples were randomly selected and rechecked with the same method.

### Statistical analysis

In the present study, SPSS software version 19.0 was employed, and the *χ*^2^ test was used to compare genotype distribution between control and case groups, as well as the distribution of demographic factors, including age, gender, educational status, occupation, family income, etc. Non-conditional logistic regression was adopted and the odds ratios (ORs) and 95% confidence intervals (95% CIs) were computed to estimate the contributions of some alleles to PTB incidence. Haplotype analysis including two and three SNPs was performed with online software (http://analysis.bio-x.cn/myAnalysis.php), and frequencies <0.03 were disregarded during analysis. The associations of the selected haplotypes with PTB incidence were estimated with ORs and 95% CIs. A two-sided *P* value <0.05 was considered to be significant.

## Results

### Distribution of demographic factors in case and control groups

A total of 168 PTB cases and 251 controls were recruited in the study. As shown in Table 2, a significant difference was detected in the distribution of age between the case and control groups (*P* = 0.030). Differences in education level and family income between the case and control groups were both significant, and the *P* values were 0.001 and 0.014, respectively. No significant differences were detected in sex, occupations, or nationalities between case and control groups.

**Table 2.** Demographic characteristics of recruited subjects

Variables	Control (*n*, %)	Case (*n*, %)	*χ*^2^	*P* value
Age			4.690	0.030
<60years	117 (46.6)	61 (35.9)		
≥60years	134 (53.4)	107 (64.1)		
Sex			0.039	0.844
Male	162 (64.5)	110 (65.5)		
Female	89 (35.5)	58 (34.5)		
Occupation			1.858	0.173
Farmer	200 (80.6)	123 (75.0)		
Others	48 (19.4)	41 (25.0)		
Nationality			0.888	0.346
Chinese Han	250 (99.6)	166 (98.8)		
Minor nationalities	1 (0.4)	2 (1.2)		
Education level			18.043	0.001
Illiteracy	64 (26.8)	64 (38.6)		
Primary school	99 (41.4)	39 (23.5)		
Junior high school	56 (23.4)	39 (23.5)		
High school	12 (5.0)	18 (10.8)		
College and above	8 (3.4)	6 (3.6)		
Average family income per year			5.985	0.014
<10 000 yuan RMB	66 (32.5)	60 (45.8)		
≥10 000 yuan RMB	137 (67.5)	71 (54.2)		

### Associations of SNP with PTB

Adjusted associations of three loci (rs1135216, rs1057141 and rs2228396) with PTB risk for age, sex and family income are shown in Table 3. No significant association was detected in the three SNPs.

**Table 3.** Associations of the SNPs with PTB

Variables	Control (*n*, %)	Case (*n*, %)	^a^Adjusted ORs (95% CIs)	*P* value
rs1135216				
AA	176 (71.3)	113 (67.7)	Reference	
AG	61 (24.7)	50 (29.9)	1.52 (0.89–2.58)	0.124
GG	10 (4.0)	4 (2.4)	0.84 (0.23–3.06)	0.792
AG/GG	71 (28.7)	54 (32.3)	1.39 (0.88–2.19)	0.156
rs1057141				
AA	151 (61.1)	104 (61.9)	Reference	
AG	73 (29.6)	57 (33.9)	1.22 (0.78–1.89)	0.382
GG	23 (9.3)	7 (4.2)	0.45 (0.19–1.10)	0.081
AG/GG	96 (38.9)	64 (38.1)	1.03 (0.68–1.56)	0.880
rs2228396				
CC	192 (76.5)	129 (76.8)	Reference	
CT	51 (20.3)	37 (22.0)	1.16 (0.71–1.90)	0.562
TT	4 (3.2)	2(1.2)	1.35 (0.21-8.49)	0.751

a Adjusted for age, sex and family income.

After stratification by age, the associations adjusting for sex and family income are shown in Table 4. A marginal significance was detected among those aged <60 years regarding the association of the rs1135216 AG/GG genotype with PTB risk (OR = 1.97, 95% CI 0.93–4.19, *P* = 0.077). No similar association was detected in other SNPs among the population aged <60 years.

**Table 4.** Associations of the SNPs with PTB among different age groups

Variables	Control (*n*, %)	Case (*n*, %)	^a^Adjusted ORs (95% CIs)	*P* value
Age < 60 years				
rs1135216				
AA	87 (75.0)	40 (66.7)	Reference	
AG	26 (22.4)	18 (30.0)	1.77 (0.81-3.87)	0.150
GG	3 (2.6)	2 (3.3)	2.76 (2.76–22.24)	0.340
AG/GG	29 (25.0)	20 (33.3)	1.97 (0.93–4.19)	0.077
rs1057141				
AA	77 (67.0)	35 (58.3)	Reference	
AG	28 (24.3)	20 (33.3)	1.92 (0.89–4.13)	0.098
GG	10 (8.7)	5 (8.3)	0.72 (0.17–3.02)	0.647
AG/GG	38 (33.0)	25 (41.6)	1.67 (0.86–3.24)	0.128
rs2228396				
CC	94 (80.3)	44 (73.3)	Reference	
CT	18 (15.4)	14 (23.3)	1.95 (0.87–4.36)	0.104
TT	3 (2.6)	2 (3.3)	2.16 (0.29–16.15)	0.453
Age ≥60 years				
rs1135216				
AA	89 (67.9)	72 (67.9)	Reference	
AG	35 (26.7)	32 (30.2)	1.30 (0.62–2.75)	0.490
GG	7 (5.3)	2 (1.9)	0.44 (0.08–2.47)	0.347
AG/GG	42 (32.0)	34 (32.1)	1.14 (0.65–2.02)	0.644
rs1057141				
AA	74 (56.1)	68 (63.6)	Reference	
AG	45 (34.1)	37 (34.5)	0.92 (0.53–1.61)	0.776
GG	13 (9.8)	2 (1.9)	0.17 (0.04–0.79)	0.024
AG/GG	58 (43.9)	39 (36.4)	0.76 (0.45–1.30)	0.316
rs2228396				
CC	98 (74.3)	85 (79.4)	Reference	
CT	33 (25.0)	22 (20.6)	0.86 (0.46–1.61)	0.643
TT	1 (0.7)	0 (0.0)	–	–
CT/TT	34 (25.8)	22 (20.6)	0.86 (0.46–1.61)	0.644

a Adjusted for sex and family income.

Among those aged ≥60 years, a significant association was detected between the rs1057141 GG genotype and increased PTB risk (OR = 0.17, 95% CI 0.04–0.79, *P* = 0.024); however, no significant association was observed in the SNPs at rs1135216 and rs2228396.

As shown in Table 5, no significant association was detected among males or females in any of the three SNPs after stratification by sex.

**Table 5.** Associations of the SNPs with PTB among male and female population

Variables	Control (*n*, %)	Case (*n*, %)	^a^Adjusted ORs (95% CIs)	*P* value
Male				
rs1135216				
AA	107 (67.3)	76 (69.1)	Reference	
AG	45 (28.3)	31 (28.2)	0.99 (0.53–1.86)	0.980
GG	7 (4.4)	3 (2.7)	0.65 (0.14–2.89)	0.566
AG/GG	52 (32.7)	34 (30.9)	1.02 (0.55–1.89)	0.962
rs1057141				
AA	90 (57.0)	67 (61.0)	Reference	
AG	54 (34.1)	38 (34.5)	0.93 (0.51–1.69)	0.807
GG	14 (8.9)	5 (4.5)	0.49 (0.14–1.71)	0.264
AG/GG	68 (43.0)	43 (39.0)	0.87 (0.49–1.53)	0.622
rs2228396				
CC	121 (76.5)	89 (80.9)	Reference	
CT	35 (22.2)	20 (18.2)	0.78 (0.39–1.55)	0.475
TT	2 (1.3)	1 (0.9)	1.99 (0.12–33.48)	0.633
CT/TT	37 (23.5)	21 (19.1)	0.81 (0.41–1.59)	0.539
Female				
rs1135216				
AA	69 (78.4)	37 (64.9)	Reference	
AG	16 (18.2)	19 (33.3)	2.10 (0.88–5.02)	0.096
GG	3 (3.4)	1 (1.8)	0.67 (0.07–6.81)	0.736
AG/GG	19 (21.6)	20 (35.1)	2.01 (0.83–4.87)	0.124
rs1057141				
AA	61 (68.5)	37 (63.8)	Reference	
AG	19 (21.3)	19 (32.8)	1.39 (0.61–3.19)	0.433
GG	9 (10.2)	2 (3.4)	4.32 (0.09–2.19)	0.311
AG/GG	18 (31.5)	21 (36.2)	1.10 (0.51–2.38)	0.803
rs2228396				
CC	71 (79.8)	40 (69.0)	Reference	
CT	16 (18.0)	17 (29.3)	1.81 (0.78–4.19)	0.165
TT	2 (2.2)	1 (1.7)	0.96 (0.08–11.29)	0.977
CT/TT	18 (20.2)	18 (31.0)	1.72 (0.76–3.86)	0.192

a Adjusted for age and family income.

### Associations of the selected haplotypes with PTB incidence

While haplotypes combined with two SNPs for analysis, the results are shown in Table 6. The results revealed that those carrying the haplotype AT at rs1057141 and rs2228396 have a significantly increased risk of PTB (OR = 2.83, 95% CI 1.30–6.14). No significant association was detected among the other haplotypes including the two SNPs.

**Table 6.** Associations of the haplotypes including two SNPs with PTB risk

Haplotype	Case (*n*, %)	Control (*n*, %)	ORs (95%CI)
rs1135216/rs2228396			
AC	249 (0.75)	386 (0.78)	0.81 (0.58–1.12)
AT	27 (0.08)	25 (0.06)	1.63 (0.93–2.86)
GC	44 (0.13)	48 (0.09)	1.40 (0.91–2.16)
GT	14 (0.04)	33 (0.07)	0.61 (0.32–1.16)
rs1057141/rs2228396			
AC	246 (0.73)	365 (0.74)	0.97 (0.71–1.33)
AT	19 (0.06)	10 (0.02)	2.83 (1.30–6.14)
GC	49 (0.15)	70 (0.14)	1.03 (0.69–1.53)
GT	22 (0.07)	49 (0.10)	0.64 (0.38–1.08)
rs1057141/rs1135216			
AA	261 (0.78)	374 (0.76)	1.16 (0.83–1.62)
GA	15 (0.04)	37 (0.08)	0.59 (0.32–1.09)
GG	56 (0.17)	81 (0.16)	1.03 (0.71–1.49)
AG	2 (0.01)	0 (0.00)	–

As shown in Table 7, when all three SNPs were included for analysis, the haplotype AAT at rs1057141/rs1135216/rs2228396 was significantly associated with increased PTB risk (OR = 2.89, 95% CI 1.34–6.27). No significant association was detected among the other haplotypes.

**Table 7.** Associations of the haplotypes including three SNPs with PTB risk

Haplotype	Case (*n*, %)	Control (*n*, %)	ORs (95% CI)
rs1057141/rs1135216/rs2228396			
AAC	242 (0.72)	364 (0.74)	0.95 (0.69–1.29)
AAT	19 (0.06)	10 (0.02)	2.89 (1.34–6.27)
GAC	8 (0.02)	21 (0.04)	0.53 (0.23–1.22)
GAT	7 (0.02)	16 (0.03)	0.66 (0.28–1.66)
GGC	41 (0.12)	49 (0.10)	1.29 (0.83–2.00)
GGT	14 (0.04)	32 (0.07)	0.66 (0.35–1.24)

## Discussion

TB is a common chronic infectious disease, which is influenced by immune status. In the present study, three SNPs located within immune regulation-related genes TAP1/2 were selected, and their contributions to PTB risk were analysed. A marginal association was detected between the rs1057141 GG genotype and PTB risk. After stratification by age, we found that the rs1057141 GG genotype carriers showed a reduced risk to PTB among those aged ≥60 years, and a marginal association was observed between the rs1135216 GG/AG genotype and PTB risk among those aged <60 years. Carriers with the haplotype AT at rs1057141 and rs2228396 or the haplotype AAT at rs1057141, rs1135216 and rs2228396 have a higher susceptibility to PTB.

TAPs are a key class of proteins that translocate antigenic peptides into the endoplasmic reticulum lumen for loading onto MHC class I molecules [9]. Since the TAP gene is closely related to antigen processing and immune regulation, the current research involving TAP mainly focuses on immune-related diseases, including one study that investigated the association of the SNP at rs1135216 of TAP1 with the onset of allergic rhinitis [17], and another study which found that the SNP at rs1057141 was associated with an increased risk of atopic disease among African populations [17]. However, no significant association was detected between either rs1057141 or rs1135216 and allergic rhinitis among the Xinjiang Han Chinese population [18]. These studies indicate that TAP-related disease incidence might be affected by ethnic characteristics and other factors.

TB is a chronic infectious disease that is considered to be affected by demographic characteristics that might cause the decline of immune status, as well as genetic factors. Recent studies revealed that rs4148871, rs4148876 and rs2857103 in TAP2 were associated with PTB risk, as well as the SNPs at rs4148871 and rs2857103 [19]. Another study conducted among the Iranian population revealed that the G allele at rs1135216 in TAP1 was significantly associated with an increased risk of TB, while the SNP at rs241447 in TAP2 was associated with a reduced risk of TB [20]. Additionally, another study conducted in South Korea also confirmed that the TAP2 gene was associated with the occurrence, development and even recurrence of TB [21]. However, no significant association was detected in another study [14].

An accumulation of previous studies revealed that age is a vital factor associated with TB incidence [22], and our present study also revealed this as an important factor, as the difference between the case and control group was significant in our study. Family income was also found to be a factor. In addition, sex was also found to be implicated in TB risk, and TB was reported to be more common in males than females, although no significance was detected in our study between the case and control groups. Thus, during statistical analysis, age, sex and family income were included as covariates in the logistic regression model to discover the real associations of genetic variants with TB.

Stratification is considered to be an effective tool to eliminate the influence of certain factors, so stratifications by sex and age were conducted in our study to find out potential associations. Our present study found that rs1135216 and rs1057141 showed no effect on PTB risk among the whole study population. However, the GG genotype at rs1057141 was associated with a reduced PTB risk among those aged 60 or more years after stratification by age. Just as previous studies reported, ageing is a key process responsible for the decline of body immunity [23], and the immune system might be disabled by the dysfunction of innate inactivation [24]. Thus, we speculate that the association of SNP at rs1057141 with PTB might be induced by age-related immune system alteration. Immunoregulation of the body is a complex process that involves the participation of many factors, including intracellular signalling mediators, which may be involved in age-related alterations in neuroendocrine-immune interactions [25]. As a result, a significant protective effect was detected among those with a reduced immune system due to increased age, while no significant association was detected among the younger population with better immune conditions. Wang *et al*. also revealed that those aged 51 years and older had a higher risk of developing TB [26].

Haplotype combination and analysis is a useful statistical analysis strategy that combines multi-loci together to discover genetic predictors of disease incidence risk [27, 28]. Our study found that both haplotypes, including AT at rs1057141 and rs2228396 and AAT at rs1057141, rs1135216 and rs2228396, were significantly associated with increased PTB risk. The results revealed that the combination of multi-site genetic variants was a useful and valuable tool to provide deep insight and more clues to discover disease incidence risk.

Our study identified that the SNP of rs1057141 was associated with decreased PTB risk among the older population, and the SNP was validated to be a functional genetic variant that could induce functional change. However, a previous study revealed that the effect could be affected by age, sex and family income, etc. Considering the distribution of the factors, we performed the analysis by adjusting for the potential factors, and consequently measured the association by stratification.

Notably, sample size might be a limit of the present study, so it will be necessary to validate the real effect of the SNPs with a study evaluating a larger sample size.

## Data Availability

The data that support the findings of this study are available on request from the corresponding author. The data are not publicly available due to privacy or ethical restrictions.
